# Bioactive Compounds and Antioxidant Capacity of *Camarosa* and *Selva* Strawberries (*Fragaria x ananassa* Duch.)

**DOI:** 10.3390/foods2020120

**Published:** 2013-03-25

**Authors:** Franco Van De Velde, Anna M. Tarola, Daniel Güemes, María E. Pirovani

**Affiliations:** 1 Food Technology Institute, Faculty of Chemical Engineering, National University of Littoral, Santiago del Estero 2829, Santa Fe 3000, Argentina; E-Mails: fvandevelde@fiq.unl.edu.ar (F.V.D.V.); dguemes@fiq.unl.edu.ar (D.G.); 2 National Council of Scientific and Technical Research (CONICET), Santiago del Estero 2829, Santa Fe 3000, Argentina; 3 Department of Management, University of Roma La Sapienza, Via del Castro Laurenziano 9, Rome 00161, Italy; E-Mail: annamaria.tarola@uniroma1.it

**Keywords:** health potential, *Camarosa* and *Selva* strawberries, antioxidant capacity, Vitamin C

## Abstract

Strawberries represent an important source of bioactive compounds due to their vitamin C and phenolic compound levels, which present high antioxidant effects, beneficial for the maintenance of consumer’s health. Argentina is the second largest strawberry producer in The Common Market of the Southern Cone (MERCOSUR), covering the main export destinations of Argentinian strawberries, *i.e.*, Canada, United States, and European Union. Information about the bioactive compound occurrence and antioxidant capacity of these fruits is scarce or not available. Health related compounds of strawberry cultivars (*Camarosa* and *Selva*) from different zones of Argentina were investigated. Vitamin C content was in the same range for both studied cultivars. However, *Camarosa* strawberries, which are the most cultivated, consumed, and exported berries in Argentina, presented higher total phenolic and anthocyanins content, and consequently better *in vitro* antioxidant capacity. Moreover, there were differences in the occurrence and concentration in the phenolic compound profiles for both cultivars. *Camarosa* cultivar presented higher content of anthocyanidins, and *Selva* showed higher total ellagic acid content. The research shows that Argentina’s strawberries are an interesting source of bioactive compounds comparable to those in other parts of the world.

## 1. Introduction

Strawberry (*Fragaria x ananassa* Duch.) is a known non-climacteric fruit of frequent human consumption with an attractive color, flavor and aroma. Strawberry is a relevant source of bioactive compounds due to its high level of vitamin C and phenolics. These compounds present antioxidant effects, and therefore a consequent beneficial effect on the maintenance of consumer health [1,2,3]. The main phenolic compounds are anthocyanins which are responsible for the fruit color, with reported concentrations of up to 65 mg/100 g fresh weight (FW) [4]. Like other fruits, strawberries can be consumed “in natura”, which is advantageous to consumers since there are no nutritional losses due to processing [1].

Argentina is the second largest strawberry producer in MERCOSUR (The Common Market of the Southern Cone), after Brazil. Coronda in Santa Fe Province (31°58′0″S, 60°55′0″W) and surroundings are one of the main producing strawberry zones in Argentina. About 60% of Argentina’s strawberries are consumed fresh, while most of the remaining production is processed, generally by individual quick freezing. The main overseas destinations of strawberries from Argentina are Canada, United States, and countries of the European Union [5]. Most of strawberries grown in the central region are of the variety “*Camarosa*” (80%), originating from the University of California, USA. It is a variety with prominent climatological adaptation, exceptional quality and flavor and a good tolerance to diseases. Furthermore, “*Selva*” variety is also cultivated in the southern regions of Argentina [6].

In order to increase the limited knowledge of Argentina’s strawberries, the aim of this study was to identify and quantify the bioactive compounds and the *in vitro* antioxidant capacity of strawberry cultivars from different regions: *Camarosa* from the central and, *Selva* from the southern region.

## 2. Experimental Section

### 2.1. Plant Material

Cultivated strawberries (*Fragaria x ananassa* Duch.) of variety *Camarosa* were obtained from one planting at Arroyo Leyes (31°27′0″S, 60°40′0″W) (Santa Fe, Argentina) during November 2009, December 2010 and November 2011. Fully developed strawberries were harvested by skilled workers at full ripe stage (90% of the surface showing a red color). They were transported 40 km directly from the field to the laboratory in Santa Fe. Strawberries (*Fragaria x ananassa* Duch.) of variety *Selva* cultivated in Mar del Plata (37°59′42″S, 57°34′0″W) (Buenos Aires, Argentina) were purchased at full ripe stage (90% surface red color) in a local market in Santa Fe, Argentina, during March 2010 and March 2011.

Physicochemical characteristics of both strawberries cultivars were: 6.6°–8.5° Brix (soluble solids), pH 3.4, and total acidity: 0.8 mg citric acid/100 g fresh weight (FW).

### 2.2. Strawberry Sample Preparation

From each lot of *Camarosa* and *Selva* strawberry received at the laboratory, 500 g were randomly sampled. Calyxes and peduncles were removed, and then fruits were washed with tap water, drained on absorbent paper and stored at −80 °C until analysis. For phenolic profile determination a portion of each strawberry cultivar was lyophilized and stored at −80 °C. The moisture was measured for strawberries by drying at 70 °C until constant weight, according to Deutsch (1995) [7]. 

### 2.3. Apparatus and Software

LC-UV vitamin C analysis was performed on a KONIK KNK-500-A Series liquid chromatograph, coupled to a variable wavelength detector (Uvis 200 Konik Instruments, Barcelona, Spain). LC-DAD phenolic compounds analysis was performed on a Shimadzu HPLC system, a LC-10AT liquid chromatograph equipped with four pumps FCV-10AL, a degasser DGU-14A, and a photodiode array detector SPD-M20A operating at wavelengths between 200 and 900 nm. LC-DAD data was processed using Shimadzu LC solution software (Shimadzu Co., Kyoto, Japan). Spectrophotometric determinations were performed with a Genesis 5 spectrophotometer with 1 cm path length cuvettes (Milton Roy, Ivyland, PA, USA). The statistical analysis of data was performed with STATGRAPHICS Centurion XV 15.2.06 (Statpoint Technologies, Inc., Warrenton, VA, USA). 

### 2.4. Chemicals and Reagents

Flavonoids (catechin, epicatechin, rutin, quercetin, kaempferol, cyanidin and pelargonidin), and phenolic acids (gallic, ferulic, coumaric and ellagic acid), DL-dithiothreitol (DTT), Folin-Ciocalteu reagent, 1,1-diphenil-2-picrylhydrazil radical (DPPH*), and 2,4,6-tripyridy-s-triazine (TPTZ) were from Sigma Chemical Co. (St. Louis, MO, USA). Metaphosphoric acid was obtained from Merck KGaA (Damnstadt, Germany). Other used chemicals and solvents were pro-analysis and were acquired from Laboratorios Cicarelli (Reagents S.A., San Lorenzo, Santa Fe, Argentina). Distilled water was purified using a Milli-Q system (Millipore, Bedford, MA, USA).

### 2.5. Ascorbic Acid and Vitamin C Content

The ascorbic acid (AA) and vitamin C (VitC) content were determined by HPLC according to van de Velde *et al.* [8]. Separations were achieved in a reversed phase column Phenomenex Gemini 5 μm C18 110A attached to a Phenomenex guardcolumn (Phenomenex Inc., Torrance, CA, USA) at 25 °C. The mobile phase, under isocratic conditions, consisted of a 0.03 mol/L sodium acetate/acetic acid buffer, 5% HPLC-grade methanol (pH = 5.8). The flow rate was 1.15 mL/min and the detection was performed at 251 nm. For extraction, samples of 5 g homogenized strawberries were added to 25 mL metaphosphoric acid (30 g/L), acetic acid (80 g/L). The mixture was homogenized for 1 min, sonicated in an ultrasonic bath for 15 min and then centrifuged at 12,000× *g* at 4 °C for 20 min. The supernatant was separated and 1 mL of it was diluted with mobile phase to achieve a final volume of 6 mL, then filtered through a 0.45 µm Millipore membrane and injected in the HPLC system for quantifying the content of ascorbic acid (AA) of the samples. For vitamin C quantification, 1 mL supernatant was added with 0.2 mL of DL-dithiothreitol solution (5 g/L DTT prepared in 2.6 mol/L potassium phosphate dibasic). The mixture was kept in the darkness at 25 °C for 2 h. Then it was made-up to volume with mobile phase to achieve a final volume of 6 mL, filtered through a 0.45 µm Millipore membrane and injected into the HPLC system. Samples were prepared in triplicates. Quantification was performed with ascorbic acid and vitamin C calibration curves and the results were expressed as mg AA or VitC/100 g FW. Dehydroascorbic acid (DHAA) content was obtained as the difference between vitamin C and ascorbic acid content.

### 2.6. Phenolic Compounds by HPLC

Phenolic compounds determination was performed by HPLC according to Tarola *et al.* [9]. The extraction was performed with 0.25 g lyophilized strawberry samples (dry weight 8.8 and 10.4 g/100 g FW in *Camarosa* and *Selva* cultivars, respectively) in 5 mL of HPLC-grade methanol. The extracts were crushed and sonicated for 15 min, then were centrifuged (2500× *g* at 25 °C for 20 min) and supernatants were collected. The insoluble plant material was re-extracted twice with 5 mL HPLC-grade methanol. Collected fractions were concentrated by a nitrogen flow at 25 °C to 5 mL and finally filtered through a Millipore 0.45 µm pore size filter. These fractions were used for the analysis of free aglycones or nonconjugated or noncondensed phenolic compounds naturally present in strawberry extracts. After taking these samples, an acid hydrolysis was performed over all obtained extracts with the objective of releasing the aglycone portion from glycosylated phenolic compounds and hydrolyzing the conjugated and condensed ones. The hydrolysis consisted in 2 mL extracted sample plus 1 mL HCl 12 N at 90 °C for 50 min. After hydrolysis, the extracts were allowed to cool and were ready to be injected in the HPLC system. Compounds were separated on a 150 mm × 4.5 mm, 5 µm particle size, Supelcosil LC-ABZ column (Sigma-Aldrich Co. LLC, St. Louis, MO, USA) and an Alltech C18 5 µm guard column (Alltech Associates Inc., Deerfield, IL, USA). The mobile phase was a gradient prepared from formic acid in water (2%, pH 3, solvent A) and formic acid in methanol (2%, pH 3, solvent B): 0.01–8.00 min, 15% B isocratic; 8.01–25.00 min, 15%–50% B; 25.01–40.00 min, 50% B isocratic; 40.01–50.00 min, 50%–90% B; 50.01–60.00 min, 90%–15% B. Ten minutes of equilibration was required before the next injection. The flow rate was 0.7 mL/min at 25 °C. The detector was set at 280, 320, 360, and 520 nm and peak identification was performed by comparison the retention times and diode array spectral characteristics with external standards and peak spiking. The quantification was performed through external standard calibration curves for catechin, epicatechin, gallic, ferulic, coumaric and ellagic acids, rutin, quercetin, kaempferol, pelargonidin, and cyanidin. Results were expressed as mg/100 g FW.

### 2.7. Antioxidant Capacity, Total Anthocyanins and Total Phenolics Content

#### 2.7.1. Extracts Preparation

For extraction, 5 g homogenized strawberries were added to 75 mL acetone/water (80:20) and sonicated for 15 min. The mixture was centrifuged at 12,000× *g* at 4 °C for 20 min and then supernatant was separated and used for analysis. 

#### 2.7.2. Total Anthocyanins Content

The total anthocyanins (TA) content was determined by the pH differential method according to Jin-Heo and Yong-Lee [10]. Each sample supernatant pH was adjusted to 1.0 with a 0.1 mol/L HCl and 25 mmol/L KCl solutions; and at pH 4.5 with a 0.4 mol/L sodium acetate/acetic acid buffer solution (triplicates). Absorbance readings were measured at 510 and 700 nm. Results were converted to mg pelargonidin-3-glucoside/100 g FW, using a molar extinction coefficient of 22,400 L/mol·cm, a molecular weight of 433.2 g/mol, an optical path of 1 cm and an absorbance (A) of:

A = [(A_510_ − A_700_)_pH 1_ − (A_510_ − A_700_)_pH 4.5_] (1)

Where A_510_ and A_700_ are the absorbance measures of samples at pH 1.0 and 4.5.

#### 2.7.3. Total Phenolics Content

The total phenolics (TP) content was determined using the Folin-Ciocalteu reagent [11]. Aliquots of 0.125 mL extracts were added with 0.25 mL of Folin-Ciocalteu reagent, 0.5 mL of Na_2_CO_3_ (20%) and 4.1 mL of distilled water. The mixture was incubated for 25 min at room temperature and then was centrifuged for 5 min at 2000× *g*. Absorbance was measured at 760 nm. Reagent blanks were prepared by replacing the sample volume by acetone/water (80:20). The total phenols content was performed by triplicates. Gallic acid was used as standard and results were expressed as mg gallic acid equivalents per 100 g FW. 

#### 2.7.4. Antioxidant Capacity by DPPH*

Antioxidant capacity was estimated by determining of the free-radical scavenging capacity evaluated with the stable radical DPPH*, according to Sánchez-Moreno *et al.* [12]. For this purpose, extract aliquots (0.025–0.075 mL) were mixed with 3.9 mL of a methanol DPPH* solution (0.03 g/L). Homogenate solutions were kept in darkness for 120 min, and absorbance was measured at 517 nm. Blanks were included replacing strawberry extracts volumes for acetone/water. The percentage of DPPH* remaining against extract concentration (mg/mL) was then plotted to obtain the amount necessary to decrease the initial DPPH* concentration by 50 % (IC_50(sample)_). The antioxidant activity of strawberry extracts were expressed as the ascorbic acid equivalent antioxidant capacity (AEAC) as mg AA/100 g FW [13]:

AEAC = IC_50(AA)_/IC_50(sample)_ × 10^5^ (2)

IC_50(AA)_ was determined with an ascorbic acid calibration curve and its value was 3.22 × 10^−3^ mg/mL.

#### 2.7.5. Antioxidant Capacity by FRAP

Antioxidant capacity was determined by means of the ferric reducing antioxidant power (FRAP) technique [14]. The FRAP was obtained by measuring the absorbance change at 593 nm caused by the reduction of the Fe^3+^-TPTZ complex to the ferrous form at pH 3.6. The FRAP reagent was freshly prepared by mixing 25 mL of acetate buffer (300 mmol/L, pH 3.6), 2.5 mL of FeCl_3_·6H_2_O solution (20 mmol/L). Briefly, 90 µL extract was added with 90 µL distilled water and 900 µL FRAP reagent. The homogenate was incubated at 37 °C for 60 min, and absorbance was measured. Blanks were included replacing strawberry extracts volumes for acetone/water. Results were expressed as mmol Fe^2+^/100 g FW, using a FeSO_4_·7H_2_O calibration curve.

## 3. Results and Discussion

### 3.1. Vitamin C Content

Vitamin C content ranged from 41.2 to 47.6 mg/100 g FW in *Camarosa* cultivar and from 28.7 to 51.0 mg/100 g FW in *Selva* cultivar. Mean values are presented in Table 1. The natural difference in the vitamin C content among strawberry cultivars was observed in previous works. A high variability in the vitamin C content was observed by Tulipani *et al.* [15] among strawberry cultivars from Italy ranging from 23 to 47 mg/100 g FW. Pincemail *et al.* [16] also describe variability among cultivars from Belgium (ranging from 33.5 to 115.7 mg/100 g FW) [7]. Vitamin C content could also vary with growing and storage conditions [17]. Ascorbic acid is the main biologically active form of vitamin C, being reversible oxidized to dehydroascorbic acid, which also exhibits biological capacity. Further oxidation generates diketogulonic acid, which has no biological function and the reaction is no longer reversible [17]. The dehydroascorbic acid content in *Camarosa* was lower than in *Selva* cultivar (Table 1). The higher percentage of DHAA in *Selva* cultivar (33.1% *vs.* 11.7% for *Camarosa* cultivar) could be due to the postharvest storage.

**Table 1:** Vitamin C (Vit C), ascorbic acid (AA), dehydroascorbic acid (DHAA), total phenolics (TP), total anthocyanins (TA), and *in vitro* antioxidant capacity (DPPH* and FRAP) in two strawberry cultivars

	Cultivar	
	*Camarosa*	*Selva*
*Bioactive compounds*		
Vit C	44.5 ± 3.2 ^a^	39.9 ± 15.8 ^a^
AA	40.2 ± 3.7 ^a^	27.5 ± 18.0 ^a^
DHAA	5.2 ± 3.7 ^a^	13.2 ± 1.6 ^b^
TP	295.3 ± 39.7 ^b^	248.4 ± 62.3 ^a^
TA	38.4 ± 10.2 ^b^	22.1 ± 4.2 ^a^
*Antioxidant capacity*		
DPPH* (AEAC)	440.1 ± 8.1 ^b^	395.7 ± 7.2 ^a^
FRAP	2.4 ± 0.5 ^b^	1.9 ± 0.9 ^a^

Vit C, AA, DHAA: mg AA/100 g FW; TP: mg gallic acid equivalents/100 g FW; TA: mg pelargonidin-3-glucoside/100 g FW; DPPH* (AEAC): ascorbic acid equivalent antioxidant capacity, mg AA/100 g FW; FRAP, mmol Fe^2+^/100 g FW; Mean ± S.D., *n=*9; Values in the same row with different superscript lowercase letters are significantly different (*p* ≤ 0.05) by *t*-test.

### 3.2. Phenolic Compounds Content

The total phenolic (TP) content and the total anthocyanin (TA) content of *Camarosa* and *Selva* strawberries are shown in Table 1. Total phenolic content (ranged from 250.0 to 324.2 mg/100 g FW) and total anthocyanin content (ranged from 27.1 to 47.0 mg/100 g FW) of *Camarosa* strawberries were higher than the values observed in *Selva* strawberries (TP ranged from 205.3 to 292.4 mg/100 g FW and TA ranged from 19.0 to 25.1 mg/100 g FW). Like vitamin C, the phenolic content in strawberries is also dependent on the cultivar and the ripening degree [1]. 

**Figure 1:**
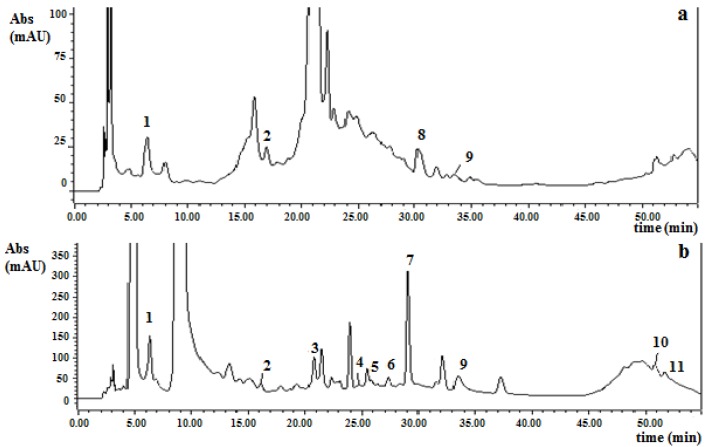
HPLC-DAD chromatograms of a *Camarosa* strawberry extract before (**a**) and after (**b**) acid hydrolysis (4 mol/L HCl, 50 min at 90 °C) at 280 nm. Peak identification: 1, gallic acid; 2, (±)-catechin; 3, (−)-epicatechin; 4, ferulic acid; 5, *p*-coumaric acid; 6, cyanidin; 7, pelargonidin; 8, rutin; 9, ellagic acid; 10, quercetin; 11, kaempferol.

**Figure 2:**
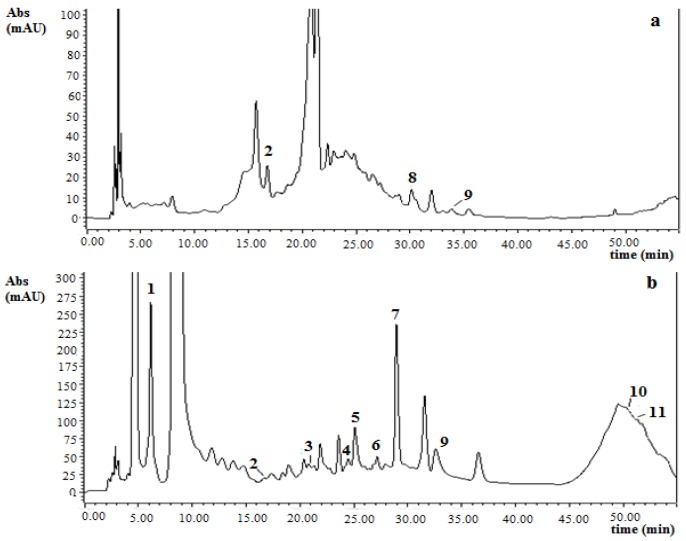
HPLC-DAD chromatograms of a *Selva* strawberry extract before (**a**) and after (**b**) acid hydrolysis (4 mol/L HCl, 50 min at 90 °C) at 280 nm. Peak identification: 1, gallic acid; 2, (±)-catechin; 3, (−)-epicatechin; 4, ferulic acid; 5, *p*-coumaric acid; 6, cyanidin; 7, pelargonidin; 8, rutin; 9, ellagic acid; 10, quercetin; 11, kaempferol.

The HPLC phenolic compounds profiles for both strawberry cultivars are shown in Table 2. Figure 1, Figure 2 illustrate a typical chromatogram at 280 nm before and after hydrolysis for *Camarosa* and *Selva* strawberries. As explained in Section 2.6, phenolic compounds obtained before acid hydrolysis represent free aglycones or nonconjugated or noncondensed phenolic compounds naturally present in the strawberry extracts. On the other hand, the phenolic compounds obtained after acid hydrolysis represent total aglycones from glycosylated phenolics or conjugated or condensed phenolic compounds in the strawberry extracts.

**Table 2:** Phenolic compounds concentration before and after acid hydrolysis in two strawberry cultivars.

Phenolic compound	Concentration before acid hydrolysis		Concentration after acid hydrolysis	
	*Camarosa*	*Selva*	*Camarosa*	*Selva*
Gallic acid	1.36 ± 0.05	-	14.28 ± 1.35 ^a^	17.21 ± 1.30 ^b^
Catechin	1.70 ± 0.17 ^a^	3.35 ± 0.42 ^b^	0.48 ± 0.09 ^a^	0.67 ± 0.10 ^a^
Epicatechin	-	-	4.02 ± 0.28 ^b^	0.84 ± 0.20 ^a^
Ferulic acid	-	-	3.44 ± 0.32 ^b^	2.75 ± 0.41 ^a^
Coumaric acid	-	-	1.69 ± 0.20 ^a^	1.96 ± 0.18 ^a^
Cyanidin	-	-	4.46 ± 0.23 ^a^	5.28 ± 0.29 ^b^
Pelargonidin	-	-	25.20 ± 1.11 ^b^	18.67 ± 1.09 ^a^
Rutin	1.0 ± 0.10 ^a^	1.84 ± 0.17 ^b^	0.09 ± 0.01 ^a^	0.10 ± 0.02 ^a^
Ellagic acid	0.61 ± 0.09 ^a^	0.65 ± 0.07 ^a^	6.67 ± 0.46 ^a^	11.87 ± 2.00 ^b^
Quercetin	-	-	0.36 ± 0.09 ^b^	0.18 ± 0.04 ^a^
Kaempferol	-	-	0.09 ± 0.02 ^a^	0.62 ± 0.09 ^b^
Total phenolics ^1^	4.66 ± 0.30 ^a^	5.82 ± 0.60 ^b^	60.79 ± 1.90 ^a^	60.20 ± 2.40 ^a^

Mean ± S.D., *n* = 9; Results expressed as mg/100 g FW; ^1^ Sum of the phenolic compound contents; -, Not detected; Different superscript lowercase letters indicate significant differences between strawberry cultivars (*p* ≤ 0.05) by *t*-test.

For *Camarosa* cultivar, gallic acid, catechin, rutin, and ellagic acid were the phenolic compounds found before acid hydrolysis (Table 2). For *Selva* cultivar, higher catechin and rutin contents were determined but gallic acid was not detected prior to hydrolysis. 

Phenolics compounds which were detected and quantified in the nonhydrolyzed strawberry extracts were lower than the content obtained after subsequent hydrolysis (Table 2). In contrast, catechin was affected by hydrolysis conditions and the content was lower in the hydrolyzed extracts. 

The remaining percentage of rutin in the hydrolyzed samples was used as a measure of the hydrolysis efficiency [9]. It was defined as the rutin concentration before acid hydrolysis minus the concentration after acid hydrolysis, divided by the concentration before acid hydrolysis. The hydrolysis efficiencies (%) were 91 % for *Camarosa* and 94.5 % for *Selva* strawberries. These results point out efficient hydrolysis in both strawberry extracts. 

Flavonols (quercetin and kaempferol) were found at low concentrations in the hydrolyzed strawberry extracts (Table 2). Although quercetin content was higher in *Camarosa* strawberries, kaempferol content was found at a lower level in this cultivar. Häkkinen and Törrönen [18] reported that quercetin ranged from 0.3 to 0.4 mg/100 g FW; and kaempferol ranged from 0.6 to 0.9 mg/100 g FW) for different strawberry cultivars from Poland and Finland. 

Concerning hydroxycinnamic acid derivatives (ferulic and coumaric acid), coumaric acid content (Table 2) was similar for both strawberry cultivars (*p* > 0.05) and was in a similar range as reported by Häkkinen and Törrönen [18] ranging from 0.7 to 1.8 mg/100 g FW in strawberries from Finland and Poland; and Mattila *et al.* [19] who reported a mean value of 2.6 mg/100 g FW in strawberries from Finland. However, ferulic acid was not reported from strawberries in these studies. In our case, ferulic acid content was 20% higher in *Camarosa* than *Selva* strawberries (Table 2).

Free ellagic acid levels (before samples hydrolysis) are generally low, although substantial quantities of this compound can be detected after acid hydrolysis of extracts, as a result of ellagitannin breakdown [2,20]. The free ellagic acid concentrations (Table 2) were similar for both studied strawberry cultivars (*p* > 0.05), and were in agreement with those reported for seven Brazilian strawberries (0.6 to 2.6 mg/100 g FW) by da Silva Pindo *et al.* [2]. Total ellagic acid content (hydrolyzed samples) was higher in *Selva* than in *Camarosa* strawberries (Table 2). The content of total ellagic acid in *Camarosa* was in agreement with the data reported by Williner *et al.* [21] for the same strawberry variety from Coronda, Argentina (mean value: 6.16 mg/100 g FW). Moreover, total ellagic content in both strawberry cultivars was slightly lower than those reported by da Silva Pinto *et al.* [2] ranging from 17 to 47 mg/100 g FW.

Anthocyanidins content can be considered as an approximate amount of the anthocyanins content (anthocyanidins in their glycoside form). In this sense, the main anthocyanins found in strawberries are pelargonidin-3-glucoside, and pelargonidin-3-rutinoside and cyanidin-3-glucoside present as minor components [2]. In this test, pelargonidin was the highest anthocyanidin found in both strawberry cultivars, with *Camarosa* concentrations being the highest (*p* ≤ 0.05). Pelargonidin content in both Argentinian strawberry cultivars (Table 2) were found to be approximately in the same range as those obtained by Giné Bordonaba *et al.* [22] who reported pelargonidin-3-glucoside concentrations from 25.4 to 40.4 mg/100 g in three strawberry cultivars (*Antea*, *Clery* and *Matis*) from Switzerland. Cyanidin concentrations obtained in this study were slightly higher than values reported by the aforementioned authors (cyanidin-3-glucoside ranged from 1.3 to 1.4 mg/100 g). Moreover, cyanidin concentration in *Selva* strawberries was higher than the value in *Camarosa* cultivar (Table 2). Total anthocyanidin contents (pelargonidin plus cyanidin) were 29.7 and 24.0 mg/100 g FW for *Camarosa* and *Selva*, respectively; which represent 48.8% and 39.8% of the sum of all phenolic compounds, respectively (Table 2). Our results were in agreement with Castro *et al.* [23] who analyzed the total anthocyanin content in *Selva* and *Camarosa* strawberries from Portugal and Spain, respectively. Authors reported that the total anthocyanin concentration in *Camarosa* was higher than in *Selva*. 

Total phenolics content was measured by the Folin-Ciocalteu method and the sum of each phenolic compound analyzed by HPLC-DAD in both strawberry cultivars. Results were different for both methodologies (Table 1, Table 2). As can be seen, total phenolics analyzed by Folin-Ciocalteu assay were higher than those obtained as the sum of phenolic compounds obtained by HPLC-DAD. It is known that Folin-Ciocalteu assay suffers from a number of interfering substances (particularly sugars, aromatic amines, sulfur dioxide, ascorbic acid, organic acid, and Fe (II)) which may also react with the reagent to give greater apparent phenolic concentrations [24]. Total phenolics content was 10% higher in *Camarosa* cultivar by the Folin-Ciocalteu method, and did not present differences between cultivars when it was calculated as the sum of phenolic compounds assayed by HPLC-DAD.

### 3.3. Antioxidant Capacity

The antioxidant activities for both methods in the cultivars are shown in Table 1. Results were in agreement with those reported by Halvonsen *et al.* [25] for three cultivars from Norway (ranging from 1.85 to 2.34 mmol Fe^2+^/100 g FW) and Leong and Shui [26], who reported a total antioxidant capacity of approximately 500 mg AA/100 g FW in strawberries from Singapore. The antioxidant capacity in *Camarosa* was higher than in *Selva* strawberries (*p* ≤ 0.05). The anthocyanins and the ellagitannins are the groups with highest contributions to total antioxidant capacity in strawberries, while it was estimated that vitamin C contributes 15% to 30% to this capacity [4,16]. The higher antioxidant capacity determined in *Camarosa* strawberries was in accordance with the higher anthocyanin content observed for these berries. However, *Selva* strawberries presented higher ellagic acid content (Table 2), which leads to a good level of antioxidant capacity in this cultivar. Since the strawberries used for this study come from different origins, it could also be possible that the differences found could be due to different culture conditions and not so much to the genetics of the cultivar.

## 4. Conclusions

The study of two strawberry cultivars from Argentina (*Camarosa* and *Selva*) indicated that there were differences in the bioactive compounds content and antioxidant capacity, but both were in the range of other varieties from others countries. The *Camarosa* cultivar presented higher total phenolic and anthocyanins content, and presented consequently a better antioxidant capacity for DPPH* and FRAP assays. However, the vitamin C content for both cultivars was in the same range. The results indicated that *Camarosa* strawberries, which are the most cultivated, consumed, and exported berries in Argentina, presented suitable bioactive compound levels with beneficial effects for human health. Moreover, the research points out that the strawberry varieties from Argentina are an interesting source of bioactive compounds comparable to those in other parts of the world.
